# Association of Podometric Parameters with the Oxford MEST-C Score and Pretreatment eGFR Slope in Patients with IgA Nephropathy

**DOI:** 10.34067/KID.0000001095

**Published:** 2025-12-18

**Authors:** Shoko Ochiai, Masao Kikuchi, Koichi Kaikita, Shouichi Fujimoto

**Affiliations:** Division of Cardiovascular Medicine and Nephrology, Department of Internal Medicine, Faculty of Medicine, University of Miyazaki, Miyazaki, Japan

**Keywords:** glomerular disease, GFR, GN, glomerulopathy, IgA nephropathy, kidney biopsy, renal biopsy

## Abstract

**Key Points:**

- Podocyte loss correlates with chronic Oxford lesions (S/T), and urinary podocyte mRNA links to active lesions (E/C).
- A lower podocyte number was the sole independent predictor of the prebiopsy eGFR slope.
- Quantitative podometrics may serve as a new biomarker to refine risk stratification in IgA nephropathy.

**Background:**

IgA nephropathy is the most prevalent primary glomerular disease worldwide; however, its heterogenous clinical course complicates prognostic prediction. Podometrics, a quantitative assessment of podocytes based on the recently proposed “podocyte depletion hypothesis,” has been suggested as a potential predictor of renal outcomes in various glomerular diseases. Nevertheless, its correlation with the Oxford classification or the prebiopsy eGFR slope remains unclear. This study aimed to investigate the association between podometrics and mesangial hypercellularity (M), endocapillary hypercellularity (E), segmental glomerulosclerosis (S), tubular atrophy/interstitial fibrosis (T), and crescent (C) scores and identify podometric parameters associated with the prebiopsy eGFR slope.

**Methods:**

Kidney biopsy specimens from 101 patients diagnosed with immunoglobulin A nephropathy at our institution between 2019 and 2022 were evaluated using the Oxford classification and podometrics. Patients were categorized into decline and nondecline groups based on their prebiopsy eGFR slope. Urinary mRNA levels of podocyte markers (NPHS1 and NPHS2) were measured in 94 patients. Independent factors associated with the decline group were identified through multivariate nominal logistic regression analysis.

**Results:**

Patients with stage S1 or T1/2 exhibited significantly lower podocyte densities and numbers compared with those with stage S0 or T0, respectively. Elevated urinary podocyte marker levels were associated with E1 and C1/C2 lesions. The decline group exhibited significantly lower podocyte density and number and larger mean podocyte volume compared with the nondecline group. In the multivariate analysis, a lower podocyte number was the only independent factor associated with the decline group.

**Conclusions:**

The podocyte number at the time of kidney biopsy was associated with the pre-biopsy eGFR decline slope in patients with immunoglobulin A nephropathy. Furthermore, elevated urinary podocyte mRNA levels suggested the presence of E and C lesions. Podometrics may serve as a potentially less invasive marker for monitoring disease activity and guiding treatment strategies.

## Visual Abstract

**Figure:**
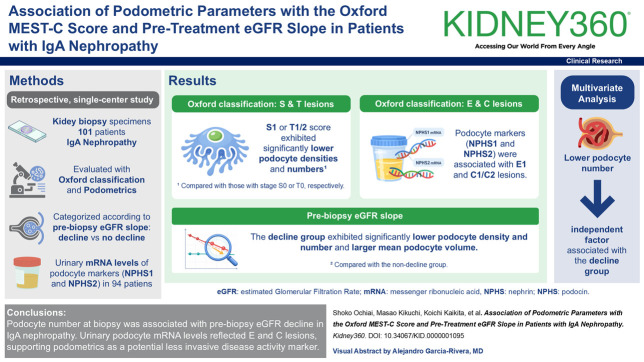

## Introduction

IgA nephropathy is the most common primary glomerular disease worldwide, with approximately 40% of patients progressing to ESKD within 20 years.^1^ Owing to its diverse clinical course, accurately predicting patient prognosis at the time of diagnosis is critically important for guiding treatment strategies. Currently, kidney biopsy remains the gold standard for diagnosing IgA nephropathy and assessing its prognosis. Histopathologic findings are evaluated using the mesangial hypercellularity (M), endocapillary hypercellularity (E), segmental glomerulosclerosis (S), tubular atrophy/interstitial fibrosis (T), and crescent (C) (MEST-C) score, as defined by the international Oxford classification.^2^ Although this classification has been shown to be useful for prognosis prediction,^3,4^ the MEST-C score is a semiquantitative assessment performed by pathologists and is, therefore, susceptible to interobserver variability.

**Figure 1:**
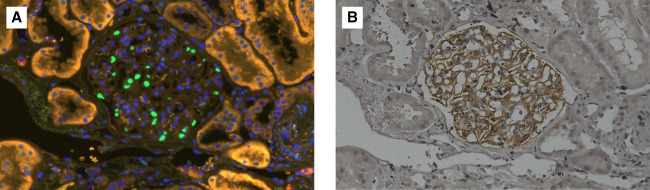
**Sequential staining of podocyte markers on the same section.** (A). TLE4 immunofluorescence image showing TLE4-positive podocyte nuclei (green) and DAPI-stained nuclei (blue). (B) The same glomerulus in the same 3 *µ*m paraffin section after immunoperoxidase staining for GLEPP1, which highlights podocyte cytoplasm. TLE4 fluorescence images were taken before GLEPP1 staining. The two images were compared by matching the glomerular outline and position to confirm that they represented the same glomerulus. The images were not digitally merged; each staining was analyzed separately. DAPI, 4',6-diamidino-2-phenylindole.

Recently, podocyte loss, as described by the podocyte depletion theory, has been proposed as a common pathway in the progression of various glomerular diseases.^5,6^ Podometrics, which measures the number and density of podocytes within glomeruli in kidney biopsy specimens, has been reported to have potential utility in predicting renal prognosis across multiple glomerular disorders.^7,8^

Furthermore, the extent of podocyte loss in the urine has been reported to correlate with renal outcomes during the clinical course,^9^ suggesting that it may inform treatment strategies for CKD characterized by long-term progression and fluctuations in disease activity. However, the relationships between podometrics and specific lesions defined by the Oxford classification remain unclear, as does the identification of parameters most strongly associated with the eGFR decline slope.

Therefore, this study aimed to characterize the association between quantitative podometric measurements and individual MEST-C scores in 101 patients with IgA nephropathy. In addition, we sought to identify podometric parameters that are independently associated with the slope of prebiopsy eGFR, a marker of disease progression.

## Methods

### Study Design and Participants

This study was conducted in accordance with the principles of the Declaration of Helsinki and approved by the Institutional Review Board of the University of Miyazaki Hospital (approval number: O-0225). Informed consent was obtained from 108 patients diagnosed with IgA nephropathy who underwent kidney biopsy at our hospital between 2019 and 2022. Of these, records from 101 patients were reviewed and analyzed, excluding seven patients for whom podometric analysis could not be performed due to inadequate immunohistochemical staining. Among the analyzed patients, 94 who provided urine samples were included in the urinary mRNA analysis.

### Clinical Data Collection and Outcome Definition

Clinical data were extracted from patients' medical records at the time of kidney biopsy. These data included age, sex, body mass index, serum creatinine, urinary protein-to-creatinine ratio (UPCR), and urinary *N*-acetyl-*β*-d-glucosaminidase-to-creatinine ratio. eGFR (ml/min per 1.73 m^2^) was calculated using the equation for Japanese adults: 194×serum creatinine^−1.094^×age^−0.287^ (×0.739 for women).^10^ We also collected data on BP, which was the measurement taken on the day of the kidney biopsy. Hypertension was defined as the use of antihypertensive medication at the time of the biopsy. The presence of diabetes mellitus and a history of gross hematuria were noted. Furthermore, the use of key medications, including renin-angiotensin-aldosterone system inhibitors and sodium-glucose cotransporter 2 inhibitors, was recorded. The prebiopsy eGFR slope was also determined by linear regression analysis using all available eGFR values during the prebiopsy period, with a minimum of three measurements required. The mean number of eGFR measurements was 3.5±0.5 times per patient, over an average observation period of 13.2±11.6 months before the kidney biopsy. Based on this slope, patients were classified into a decline group (negative slope) and a nondecline group (zero or positive slope).

### Histopathologic Assessment and Podometrics

All kidney biopsy specimens were independently assessed by two experienced pathologists according to the Oxford classification (MEST-C score). Podometric analysis was performed based on the method described by Venkatareddy *et al*.^11^ In brief, podocyte nuclei were stained with a monoclonal antibody against TLE4 (Santa Cruz Biotechnology, cat. no. SC-365406, 1:150 dilution) *via* immunofluorescence. In addition, podocyte cytoplasm was subsequently stained on the identical 3-*µ*m thick section using the GLEPP1 immunoperoxidase method with a rabbit polyclonal anti-GLEPP1 antibody (Abcam, cat. no. MABS1221, 1:750 dilution). The TLE4 immunofluorescence images and the GLEPP1 bright-field images of the same glomerulus were visually matched by comparing their shape and anatomical architecture to confirm colocalization of TLE4-positive nuclei within GLEPP1-positive cytoplasm (Figure 1). The two images were analyzed separately without digital merging. Measurements were performed using a Keyence BZ-X810 fluorescence microscope. Podometric parameters, including podocyte density and number, were calculated using previously reported formulas.^9^ These calculations incorporated variables such as the number of observed podocyte nuclei, mean nuclear diameter, the section thickness of 3 *µ*m, and tuft area. Glomerular volume was estimated using the Weibel and Gomez method.^12^ All glomeruli within each 3-*μ*m thick section were analyzed, with an average of 26.9±10.6 glomeruli evaluated per biopsy.

### Urinary mRNA Analysis

Morning urine samples were collected and centrifuged at 3200 *g* for 15 minutes at 4°C. The resulting pellet was washed, resuspended in RLT buffer (Qiagen)/*β*-mercaptoethanol buffer (RNeasy Kit, Qiagen), and stored at −80°C until further use. Total RNA was extracted from the urine pellets using an RNeasy Mini Kit (Qiagen, Hilden, Germany, cat. no. 74104). The cDNA was subsequently synthesized using a High-Capacity cDNA Reverse Transcription Kit (Applied Biosystems, Thermo Fisher Scientific, Waltham, MA, cat. no. 4368814).

The mRNA levels of target genes, including NPHS1 and NPHS2, were quantified by real-time PCR using a Lightcycler96 system (Roche, Basel, Switzerland) with TaqMan probes and TaqMan Fast Universal PCR Master Mix in a total reaction volume of 10 *μ*l. The thermal cycling conditions were as follows: initial denaturation at 95°C for 10 minutes, followed by 40 cycles of 95°C for 15 seconds and 60°C for 1 minute. All measurements were performed in duplicate using 1 *μ*g of sample cDNA per reaction and quantified against cDNA standards. The final mRNA levels were normalized to urinary creatinine concentration.

The following TaqMan probes (Applied Biosystems) were employed: human NPHS1 (nephrin, cat. no. Hs00190446_m1) and human NPHS2 (podocin, cat. no. Hs00922492_m1).

### Statistical Analyses

All statistical analyses were performed using JMP software (SAS Institute Inc., Cary, NC). Continuous variables are expressed as the mean±SD, and categorical variables are expressed as numbers (%). Comparisons between two groups were performed using Welch *t* test, and correlations were assessed using Spearman rank correlation coefficient. Multivariate nominal logistic regression analysis was used to identify independent factors associated with eGFR decline. Multivariate models were adjusted for clinically relevant potential confounders, including age, sex, eGFR at biopsy, and proteinuria. Statistical significance was set at a *P* value of <0.05.

## Results

### Patient Characteristics

The analysis included 101 patients with kidney biopsy-proven IgA nephropathy. The baseline clinical and pathologic characteristics are summarized in Table 1. The mean age of the patients was 42.9±15.9 years, and 39.6% (*n*=40) were men. The mean eGFR at the time of biopsy was 69.8±27.1 ml/min per 1.73 m^2^, and the mean UPCR was 1.36±1.79 g/gCr. An average of 26.9±10.6 glomeruli was analyzed per biopsy. According to the Oxford classification, the prevalence rates of mesangial hypercellularity (M1), endocapillary hypercellularity (E1), and segmental glomerulosclerosis (S1) were 16.8%, 39.6%, and 72.3%, respectively. The prevalence of tubulointerstitial fibrosis (T) lesions was 6.0% (T1=3.0% and T2=3.0%), and the prevalence of cellular/fibrocellular crescent (C) lesions was 51.5% (C1=49.5% and C2=2.0%).

**Table 1:** Baseline characteristics

Characteristics	Total patient (*n*=101)
Age (y.o.)	42.9±15.9
Sex male, *n* (%)	40 (39.6%)
BMI	23.8±4.0
sCre (mg/dl)	0.96±0.48
eGFR (ml/min per 1.73 m^2^)	69.8±27.1
UPCR (g/gCr)	1.36±1.79
Gross hematuria history, *n* (%)	40 (39.6%)
**Severity of hematuria**	
(−), *n* (%)	1 (1.0%)
(±), *n* (%)	8 (7.9%)
(1+), *n* (%)	9 (8.9%)
(2+), *n* (%)	24 (23.8%)
(3+), *n* (%)	59 (58.4%)
**BP (mm Hg)**	
Systolic/Diastolic	122.5±15.5/75.0±11.9
Hypertension (on medication), *n* (%)	45 (44.6%)
Diabetes mellitus, *n* (%)	6 (5.9%)
RAAS inhibitor use, *n* (%)	38 (37.6%)
SGLT2 inhibitor use, *n* (%)	4 (4.0%)
Number of glomeruli (*n*)	26.9±10.6
Global sclerosis (%)	3.1±3.2(13.1%±13.8)
Oxford classification MEST-C score	
M1, *n* (%)	17 (16.8%)
E1, *n* (%)	40 (39.6%)
S1, *n* (%)	73 (72.3%)
T1/T2, *n* (%)	3 (3.0%)/3 (3.0%)
C1/C2, *n* (%)	50 (49.5%)/2 (2.0%)
Glomerular volume (×10^6^ *μ*m^3^)	2.78±0.93
Podocyte density (/10^6^ *μ*m^3^)	151.1±51.0
Podocyte number (/glomurulus)	362.1±110.0
Podocyte volume (*μ*m^3^)	3457±1366

Baseline clinical, histologic, and podometric characteristics. Data are presented as mean±SD or *n* (%). BP was measured on the day of kidney biopsy. Hypertension was defined as the use of antihypertensive medication. BMI, body mass index; MEST-C, mesangial hypercellularity (M), endocapillary hypercellularity (E), segmental glomerulosclerosis (S), tubular atrophy/interstitial fibrosis (T), and crescent (C); RAAS, renin-angiotensin-aldosterone system; sCre, serum creatinine; SGLT2, sodium-glucose cotransporter 2; UPCR, urinary protein-to-creatinine ratio.

### Association between Oxford MEST-C Score and Clinicopathologic/Podometric Parameters

The associations between each component of the Oxford classification and various clinicopathologic and podometric parameters were analyzed (Table 2). eGFR was associated only with T lesions, being significantly lower in T1 compared with T0 (eGFR T0: T1=72.0±26.3:36.2±12.8, *P* = 0.0003). Urinary protein/urinary creatinine ratio (UP/UCr) was higher in M0, S1, and C1/2 compared with that in M1, S0, and C0, respectively, but did not differ based on the presence or absence of E or T lesions. Similarly, urinary *N*-acetyl-*β*-d-glucosaminidase-to-creatinine ratio was increased in M1, S1, and C1/2 compared with that in M0, S0, and C0, respectively.

**Table 2:** Associations between Oxford MEST-C score and clinicopathologic/podometric parameters

Parameter	M0 (*n*=84)	M1 (*n*=17)	Univariate *P* Value	E0 (*n*=61)	E1 (*n*=40)	Univariate *P* Value	S0 (*n*=28)	S1 (*n*=73)	Univariate *P* Value	T0 (*n*=95)	T1 and T2 (*n*=6)	Univariate *P* Value	C0 (*n*=49)	C1 and C2 (*n*=52)	Univariate *P* Value
Clinical and biochemical variables															
Age (y.o.)	44.2±16.1	36.5±14.1	0.0580	44.0±15.5	41.2±16.7	0.4115	43.7±16.1	42.6±16.0	0.7563	42.0±15.4	56.3±19.2	0.1288	43.1±15.7	42.7±16.3	0.8884
eGFR (ml/min per 1.73 m^2^)	69.3±27.2	72.5±27.3	0.6686	67.6±23.9	73.3±31.3	0.3319	75.3±23.8	67.8±28.1	0.1825	72.0±26.3	36.2±12.8	0.0003^a^	70.0±26.6	69.7±27.7	0.9530
UPCR (g/gCr)	1.46±1.92	0.91±0.71	0.0480^a^	1.20±1.69	1.61±1.92	0.2698	0.94±0.94	1.53±2.00	0.0464^a^	1.23±1.48	3.56±4.10	0.2234	0.87±1.01	1.83±2.20	0.0056^a^
UNAG/UCr (U/gCr)	0.091±0.067	0.069±0.030	0.0486^a^	0.080±0.055	0.098±0.073	0.2083	0.066±0.037	0.096±0.069	0.0086^a^	0.083±0.061	0.154±0.071	0.0898	0.066±0.042	0.106±0.072	0.0013^a^

Parameter	M0 (*n*=84)	M1 (*n*=17)	Univariate *P* Value	Multivariate *P* Value	E0 (*n*=61)	E1 (*n*=40)	Univariate *P* Value	Multivariate *P* Value	S0 (*n*=28)	S1 (*n*=73)	Univariate *P* Value	Multivariate *P* Value
Podometric variables												
Glomerular volume (×10^6^ *μ*m^3^)	2.79±0.94	2.79±0.87	0.9797	0.5703	2.84±1.06	2.70±0.67	0.4068	0.6249	2.56±0.88	2.87±0.94	0.1251	0.1554
Podocyte density (/10^6^ *μ*m^3^)	152.5±51.8	144.1±47.9	0.5235	0.1677	153.6±54.4	147.3±45.8	0.5317	0.2060	173.5±54.6	142.5±47.1	0.0110^a^	0.0053^a^
Podocyte number (/glomurulus)	366.0±115.8	343.1±74.5	0.3074	0.2731	364.3±103.0	358.8±121.2	0.8128	0.5008	391.1±105.6	351.0±110.4	0.0981	0.1540
Podocyte volume (*μ*m^3^)	3456.5±1406.6	3462.3±1181.2	0.9859	0.3672	3575.0±1455.5	3278.2±1212.2	0.2697	0.4897	3317.1±1537.5	3511.3±1301.5	0.5572	0.5232

Parameter	T0 (*n*=95)	T1 and T2 (*n*=6)	Univariate *P* Value	Multivariate *P* Value	C0 (*n*=49)	C1 and C2 (*n*=52)	Univariate *P* Value	Multivariate *P* Value
Glomerular volume (×10^6^ *μ*m^3^)	2.79±0.93	2.81±0.92	0.9490	0.2474	2.89±0.10	2.69±0.80	0.2916	0.1961
Podocyte density (/10^6^ *μ*m^3^)	153.8±50.7	108.6±36.7	0.0278^a^	0.5420	146.9±50.2	155.1±51.9	0.4226	0.3013
Podocyte number (/glomurulus)	369.8±108.6	240.5±39.8	<0.0001^a^	0.0445^a^	360.7±106.5	363.5±114.3	0.8999	0.9587
Podocyte volume (*μ*m^3^)	3433.1±1376.4	3842.5±1225.8	0.4619	0.4659	3784.1±1470.5	3149.6±1193.1	0.0197^a^	0.0070^a^

Data are presented as mean±SD. *P* values for the univariate analysis were calculated using Welch’s *t* test. Multivariate *P* values were obtained using a multivariate model adjusted for age, sex, and eGFR at biopsy. MEST-C, mesangial hypercellularity (M), endocapillary hypercellularity (E), segmental glomerulosclerosis (S), tubular atrophy/interstitial fibrosis (T), and crescent (C); UNAG/UCr, urinary *N*-acetyl-*β*-d-glucosaminidase-to-creatinine ratio; UPCR, urinary protein-to-creatinine ratio.

a *P* < 0.05.

Analysis of podometric parameters revealed that glomerular podocyte density was significantly lower in S1 than in S0 and was negatively correlated with the percentage of glomeruli exhibiting segmental sclerosis (*r*=−0.2180, *P* = 0.0285). The total number of podocytes per glomerulus was significantly reduced in T1/2 than in T0. In addition, the mean podocyte volume was significantly lower in C1/2 than in C0 and was negatively correlated with the percentage of glomeruli containing crescents (*r*=−0.2207, *P* = 0.0266). These associations remained significant in multivariate models adjusted for age, sex, and eGFR at the time of biopsy.

### Association of Podometric Parameters with the Slope of Pretreatment eGFR

The mean prebiopsy eGFR slope in this cohort was −14.68±51.78 ml/min per 1.73 m^2^/year. Among the 101 patients, 70 (69.3%) exhibited a negative eGFR slope (decline group), whereas 31 (30.7%) showed no decline in eGFR slope (nondecline group).

Several baseline differences were observed between the two groups (Table 3). Compared with the nondecline group, patients in the decline group were significantly older (*P* = 0.0001) and had a lower eGFR at biopsy (*P* < 0.0001). They also exhibited significantly higher UPCR (*P* = 0.0224) and urinary N-acetyl-β-D-glucosaminidase-to-creatinine ratio (*P* < 0.0001). As regards podometric parameters, the decline group demonstrated significantly lower podocyte density (*P* = 0.0263) and number (*P* = 0.0149) and a significantly larger mean podocyte volume (*P* = 0.0220).

**Table 3:** Baseline characteristics of patients grouped by prebiopsy eGFR slope

Parameters	Nondecline (*n*=31)	Decline (*n*=70)	*P* Value
Age (y.o.)	33.0±16.7	47.2±13.5	<0.0001
BMI	22.7±3.6	24.2±4.1	0.0635
sCre (mg/dl)	0.70±0.14	1.07±0.53	<0.0001
eGFR (ml/min per 1.73 m^2^)	92.3±24.9	59.9±21.6	<0.0001
UPCR (g/gCr)	0.89±0.97	1.58±2.02	0.0224
UNAG/UCr	0.055±0.032	0.101±0.008	<0.0001
Global sclerosis, *n* (%)	1.8±1.9 (6.8%±7.5)	3.7±3.5 (15.9%±15.0)	0.0007

Parameters	Nondecline (*n*=31)	Decline (*n*=70)	*P* Value
Glomerular volume (×10^6^ *μ*m^3^)	2.66±0.78	2.85±0.99	0.2988
Podocyte density (/10^6^ *μ*m^3^)	166.9±43.7	144.1±52.7	0.0263
Podocyte number (/glomerulus)	400.0±96.1	345.3±112.2	0.0149
Podocyte volume (*μ*m^3^)	3048.1±1017.0	3638.7±1464.7	0.0220

Data are presented as mean±SD. Comparisons between two groups were performed using Welch *t* tests. BMI, body mass index; sCre, serum creatinine; UNAG/UCr, urinary N-acetyl-b-d-glucosaminidase-to-creatinine ratio; UPCR, urinary protein-to-creatinine ratio

The association between individual Oxford scores and the prebiopsy eGFR slope was also examined. Among the MEST-C components, patients with T1/2 lesions had a significantly more negative eGFR slope compared with those with T0 lesions (*P* = 0.0008). No significant associations were found between the M, E, S, or C scores and eGFR slope (Table 4).

**Table 4:** Associations between Oxford classification and pre-biopsy eGFR slope

Oxford Score	eGFR Slope (mean±SD)	Univariate *P* Value	Multivariate *P* Value
M0 (*n*=84)	−17.2±56.3	0.0254	0.1608
M1 (*n*=17)	−2.1±10.5		
E0 (*n*=61)	−12.3±56.6	0.5491	0.5151
E1 (*n*=40)	−18.3±6.9		
S0 (*n*=28)	−2.8±23.4	0.0471	0.5021
S1 (*n*=73)	−19.2±58.7		
T0 (*n*=95)	−9.7±32.4	0.2594	0.0008*
T1 and T2 (*n*=6)	−93.4±161.1		
C0 (*n*=49)	−11.1±9.2	0.5098	0.9467
C1 and C2 (*n*=52)	−18.1±36.7		

Data are expressed as the mean±SD (ml/min per 1.73 m^2^/year). Comparisons between the two groups were performed using Welch *t* tests. C, cresent; E, endocapillary hypercellularity; M, mesangial hypercellularity; S, segmental glomerulosclerosis; T, tubular atrophy/interstitial fibrosis.

In the subsequent multivariate logistic regression analysis, a lower podocyte number was identified as the sole independent factor associated with eGFR decline (Table 5). When the MEST-C components (M, E, S, T, and C) were included in the multivariable logistic regression model along with the podocyte number, both the podocyte number (*P*= 0.053) and the S component (*P* = 0.058) showed a trend toward association with the decline group, whereas the remaining MEST-C components were not significant (Table 6).

**Table 5:** Multivariate logistic regression analysis of factors associated with prebiopsy eGFR decline group

Variable	Likelihood Ratio Chi squared	*P* Value
Glomerular volume (×10^6^ *μ*m^3^)	3.13	0.0769
Podocyte density (/10^6^ *μ*m^3^)	1.86	0.1723
Podocyte number (/glomerulus)	4.93	0.0263
Podocyte volume (*μ*m^3^)	0.003	0.9564

A multivariate logistic regression model was constructed including all podometric variables listed in the table to assess their independent associations with eGFR decline. Effects of each variable was adjusted for all other variables in the model.

**Table 6:** Associations of histologic and podometric factors with prebiopsy eGFR decline

Variable	Likelihood Ratio Chi squared	*P* Value
Podocyte number	3.75	0.0529
S score (S1 versus S0)	3.61	0.0575
M score (M1 versus M0)	1.94	0.1635
E score (E1 versus E0)	1.83	0.1764
T score (T1 and 2 versus T0)	1.51	0.2188
C score (C1 and 2 versus C0)	0.82	0.3644

A multivariate logistic regression model was constructed, including the podocyte number and Oxford classification (MEST-C) scores. Effect of each variable was adjusted for all other variables in the model. MEST-C, mesangial hypercellularity (M), endocapillary hypercellularity (E), segmental glomerulosclerosis (S), tubular atrophy/interstitial fibrosis (T), and crescent (C).

### Association of Urinary mRNA Markers with Histologic Lesions

Analysis of urinary mRNA markers revealed specific associations with histologic lesions (Table 7). In multivariate models adjusted for age, sex, and eGFR, E1 lesions were independently associated with significantly higher urinary NPHS1/Cre levels (*P* = 0.0028). Similarly, C1 and C2 lesions were independently associated with elevated NPHS1/Cre (*P* = 0.0240) and NPHS2/Cre levels (*P* = 0.0199). By contrast, no significant association was observed between the presence of M, S, or T lesions and urinary podocyte mRNA markers. Correlation analysis further supported these findings. A higher percentage of crescents (cres%) was positively correlated with both NPHS1/Cre (*r*=0.2575, *P* = 0.0122) and NPHS2/Cre (*r*=0.3828, *P* = 0.0001). Similarly, a higher percentage of endocapillary hypercellularity (end%) was positively correlated with both NPHS1/Cre (r=0.2988, *P* = 0.0034) and NPHS2/Cre (*r*=0.2969, *P* = 0.0037). Regarding the relationship between urine podometrics and biopsy podometrics, we observed weak negative correlations between urinary nephrin and podocin mRNA and glomerular volume (*P* = 0.075, 0.090, respectively), and between urinary podocin mRNA and podocyte number (*P* = 0.098), but these were not statistically significant.

**Table 7:** Associations between Oxford classification and urinary mRNA markers

mRNA Markers	Oxford Classification		Univariate *P* Value	Multivariate *P* Value
	M0 (*n*=78)	M1 (*n*=16)		
NPHS1/Cre	1	1.97±1.15	0.7947	0.9910
NPHS2/Cre	1	1.13±1.30	0.7373	0.7977

	E0 (*n*=57)	E1 (*n*=37)		
NPHS1/Cre	1	3.03±4.89	0.0182^a^	0.0028^a^
NPHS2/Cre	1	1.43±1.69	0.2597	0.2666

	S0 (*n*=28)	S1 (*n*=66)		
NPHS1/Cre	1	1.36±2.53	0.4578	0.6339
NPHS2/Cre	1	1.60±2.53	0.1258	0.1889

	T0 (*n*=89)	T1 and T2 (*n*=5)		
NPHS1/Cre	1	0.40±0.43	0.0439	0.3561
NPHS2/Cre	1	0.65±0.48	0.2240	0.7067

	C0 (*n*=46)	C1 and C2 (*n*=48)		
NPHS1/Cre	1	2.49±4.28	0.0273^a^	0.0240^a^
NPHS2/Cre	1	2.07±2.91	0.0307^a^	0.0199^a^

Data are expressed as the mean±SD. *P* values for the univariate analysis were calculated using Welch *t* test. *P* values in parentheses represent the results of the multivariate analysis adjusted for age, sex, and eGFR at biopsy. C, cresent; E, endocapillary hypercellularity; M, mesangial hypercellularity; S, segmental glomerulosclerosis; T, tubular atrophy/interstitial fibrosis.

a *P* < 0.05.

## Discussion

Podocytes are terminally differentiated, long-lived cells that are not readily replaced. They reside on the outer surface of the glomerular basement membrane; when they detach, they are excreted in the urine, resulting in permanent loss. Indeed, podocyte numbers also decrease with age.^13,14^ Recently, the podocyte depletion hypothesis has been proposed, suggesting that podocyte loss triggers a cascade of events leading to glomerulosclerosis and, ultimately, ESKD. Consequently, quantitative assessment of podocytes may be critical for predicting renal prognosis. The quantitative podocyte assessment employed in this study, termed podometrics, comprises two approaches: biopsy podometrics and urine pellet podometrics.^7^ Biopsy podometrics includes parameters such as the number of podocytes within the glomerulus, their mean volume, and their density. These parameters change with aging, with podocyte number decreasing, mean volume increasing, and density declining.^13,14^ Moreover, these parameters have been reported to correlate strongly with eGFR.^15^ Furthermore, podocyte detachment can be monitored noninvasively through urine pellet podometrics. Wickman *et al*. reported elevated urinary podocyte mRNA levels in patients with various glomerular diseases compared with those in normal controls,^8^ and Naik *et al*. demonstrated that the extent of podocyte detachment in the urine of kidney transplant recipients can predict donor kidney prognosis.^16^

Based on these findings, this study used the Oxford classification, a widely used pathologic assessment method for IgA nephropathy, to examine how the presence or absence of each component correlates with podometrics.

Clinical parameters most strongly correlated with renal prognosis in glomerular diseases, including UP/UCr and eGFR, are well established. Our results demonstrated that the UP/UCr ratio was associated with M, S, and C lesions, whereas eGFR was associated only with T lesions. This pattern reflects the typical progression of IgA nephropathy, in which microscopic hematuria develops into proteinuria, ultimately leading to impaired renal function. T lesions are considered indicative of advancing renal impairment.

Analysis of biopsy podometrics revealed that the presence of S or T lesions was associated with reduced podocyte density or number within the glomerulus. Given that decreased podocyte density or number is a central feature of the podocyte depletion hypothesis, these findings underscore the pathologic significance of S and T lesions in IgA nephropathy progression.

S lesions are well recognized as predictors of poor outcomes in IgA nephropathy, as confirmed by numerous large studies, and their underlying causes can be heterogeneous.^2,3^ The Oxford Working Group has emphasized that S lesions exhibiting signs of podocyte injury are particularly important, as these features are associated with increased proteinuria and more rapid decline in kidney function.^2^ Our analysis supports this observation, demonstrating a clear negative correlation between the extent of segmental sclerosis and podocyte number. This finding aligns with the study by Lemley *et al*., who initially reported an association between podocytopenia and disease severity in patients with IgA nephropathy.^17^ The association with T lesions is also of critical significance. A recent systematic review confirmed that tubulointerstitial fibrosis represents the most robust prognostic predictor within the Oxford classification and is significantly associated with adverse outcomes in 58% of the analyses.^18^ Our results provide a potential pathologic basis for this clinical observation. Notably, podometrics—despite being confined to podocytes within the glomeruli—showed a strong association with T lesions. Inflammatory cell infiltration and interstitial fibrosis surrounding sclerotic glomeruli are commonly observed in kidney biopsy specimens. This finding demonstrates an association between glomerular and tubulointerstitial lesions.

Furthermore, podocyte volume was smaller in C1 than in C0. Previous studies have indicated that podocytes undergo hypertrophy as an adaptive response to cover the glomerular tuft when podocyte numbers decline. The rapid process leading to crescent formation suggests that this adaptive mechanism may not have kept pace. However, impaired adaptive capacity of the patient's podocytes may have triggered the crescent formation.

In this study, no differences were found in eGFR or UP/UCr between the E0 and E1 groups, and biopsy podometrics revealed no differences in podocyte number, mean volume, or density. However, the presence of E lesions was associated with an approximately three-fold increase in urinary nephrin mRNA levels. Previous studies from our department similarly demonstrated increased urinary podocin mRNA levels in the presence of E lesions,^19^ suggesting a link between E lesions and podocyte loss. Although this study did not find an association between E lesions and urinary podocin mRNA, urinary detachment of podocin and nephrin differs according to disease stage,^20^ which may explain the discrepancy. The prognostic significance of E lesions has been debated, partly due to treatment bias in retrospective cohorts. However, recent studies in untreated patients have identified E lesions as important predictors of progression.^21^ These findings may provide a relevant clinical parameter supporting this finding.

Recently, eGFR slope has gained prominence as a key predictor of renal prognosis. In this study, patients were divided into two groups based on their pre-biopsy eGFR slope (progressive or nonprogressive) to examine their association with the Oxford classification and podometrics. Biopsy podometrics revealed that podocyte numbers were decreased in patients exhibiting a prebiopsy eGFR decline. Consistent with the Oxford classification, the presence of T lesions was associated with a rapid decline in eGFR. These findings align with those of Denic *et al*., who identified a lower absolute podocyte number as an independent predictor of renal disease progression in patients with progressive CKD.^22^ As kidney disease often presents with few subjective symptoms, renal function measurements may not have been performed at least three times before biopsy in some patients. In such patients, urinary nephrin and podocin mRNA may serve as important alternative markers.

This study has some limitations, including its retrospective single-center design and relatively small sample size. To validate these findings and facilitate clinical application, large multicenter prospective studies are warranted.

In summary, the results of this study indicate that podocyte number and the presence of S lesions at the time of kidney biopsy in IgA nephropathy are associated with the pre-biopsy eGFR decline slope. Furthermore, elevated urinary podocyte mRNA levels reflected the presence of E and C lesions. In CKD such as IgA nephropathy, even after formulating and implementing a treatment plan based on biopsy findings, it remains crucial to reassess the treatment strategy while monitoring treatment response to optimize efficacy. Although a repeat kidney biopsy can provide valuable information, it is invasive and carries the risk of serious complications. Therefore, less invasive and more practical markers are desirable, and podometrics may serve as a promising alternative.

## Data Availability

Original data generated for the study will be made available upon reasonable request to the corresponding author. Data Type: Observational Data. Reason for Restricted Access: The datasets generated and analyzed during the current study are not publicly available due to ethical restrictions as they contain information that could compromise participant privacy. This restriction has been imposed by the Institutional Review Board of Miyazaki University Hospital. However, data are available from the corresponding author upon reasonable request, subject to a data sharing agreement and approval by the aforementioned ethics committee.

## Declarative Statements

This study includes clinical experimentation and received Institutional Review Board or Ethics Committee approval. All patients provided written informed consent. This study includes clinical experimentation and complies with the Declaration of Helsinki.
